# Editorial: Investigating Antimicrobial Resistance With Single-Molecule Sequencing Technologies: Opportunities and Challenges

**DOI:** 10.3389/fmicb.2022.913662

**Published:** 2022-05-12

**Authors:** Ruichao Li, Mashkoor Mohsin, Nilton Lincopan

**Affiliations:** ^1^Jiangsu Co-innovation Center for Prevention and Control of Important Animal Infectious Diseases and Zoonoses, College of Veterinary Medicine, Yangzhou University, Yangzhou, China; ^2^Institute of Microbiology, University of Agriculture, Faisalabad, Pakistan; ^3^Department of Microbiology, Institute of Biomedical Sciences, Universidade de São Paulo, São Paulo, Brazil

**Keywords:** antimicrobial resistance, microbial genomics, long-read sequencing, mobile genetic elements, single-molecule analysis

The emergence, dissemination, and evolution of antimicrobial-resistant pathogens among animals, humans, and the environment have caused serious public health concern and is exacerbating globally (Hernando-Amado et al., 2019; Antimicrobial Resistance Collaborators, 2022). Horizontal gene transfer is mainly responsible for the widespread transmission of traditional and novel antimicrobial resistance (AMR) genes by mobile genetic elements including plasmids, transposons, integrative and conjugative elements, and insertion sequences (Partridge et al., 2018; Arnold et al., 2021). Next-generation sequencing technologies along with high-quality bioinformatics tools have contributed to a more refined investigation of AMR research in terms of genomic epidemiology and rapid detection (Ashton et al., 2015; Gu et al., 2020).

However, the diversity and evolution of mobile genetic elements are impossible to resolve with only short-read next-generation sequencing technologies because of the complex repetition of MDR regions and polymorphism of such elements. Long-read and single-molecule sequencing technologies such as PacBio single-molecule real-time (SMRT) or Oxford Nanopore Sequencing (ONT) are increasingly used to tackle problems in the field of AMR impossible to be addressed before. Challenges with such sequencing technologies also exist, exemplified by the low accuracy of raw long-reads, relatively low-throughput, and high economic cost.

The six articles published in this Research Topic covered the different applications of long-read sequencing methods and launched the first Qitan Nanopore sequencing in microbial genomics. In this regard, long-read sequencing technology offers irreplaceable and significant advantages in genome assembly, large structural variation detecting, complex population analysis, and so on. The current third-generation long-read sequencing is dominated by PacBio SMRT and ONT sequencing technologies (Amarasinghe et al., 2020). Recently, a novel nanopore sequencing technology developed by QitanTech is developing rapidly, which is the first long-read sequencer released in China. Peng et al. participated in the Early User Program of QitanTech and evaluated its performance in resolving accurate genome structures of important AMR bacteria-harboring *tet*(X), *tmexCD*-*toprJ*, and *bla*_VIM−2_, aiming to evaluate the ability of QitanTech long-read sequences in assembling complete bacterial genomes with different assembly strategies. They found that using QitanTech nanopore sequencing data, most MDR bacterial genome structures could be well-resolved successfully including cointegrate plasmids, although high accurate Illumina short-read data is necessary to avoid indel errors. The feasibility of the novel long-read sequencing platform in microbial genomics study will benefit AMR research in an unprecedented way. Although ONT nanopore sequencing is increased in raw read accuracy (Wang et al., 2021), it is still difficult to catch up with second-generation sequencing within a short period of time. As a result, both ONT nanopore sequencing and QitanTech nanopore sequencing technologies remain in need of improvements, especially in sequencing accuracy.

Bird et al. utilized ONT nanopore sequencing to resolve accurate genomic locations of *bla*_CTX−M−15_ and found that *bla*_CTX−M−15_-encoding plasmids, as well as chromosomal integration events, existed in *E. coli* strains from fecal microbiota samples in travelers. In the USA, Li X. et al. characterized 134 *Salmonella* isolates of multiple serotypes recovered from food products by the NARMS across 31 different states between 2016 and 2018 with PacBio long-read sequencing, and explored resistome, virulence genes, pathogenicity islands arrangement, and evolution, including chromosome integration of plasmid sequences and serotype switching from S. Typhimurium to *S*. 4,[5],12:i:-. The authors reported variation in *Salmonella* pathogenicity islands, genomic islands, and integrated plasmids, which resulted in genomic variability among isolates. This study advocates the importance of AMR surveillance using long-read sequencing and also expands the repository of complete reference genomes of *Salmonella* species.

Boostrom et al. performed a systematic evaluation of different assembly strategies of ONT nanopore long-read sequencing data of selected bacterial isolates and compared their accuracies to recover mobile genetic elements especially antibiotic-resistance genes. Even with long-read data, misinterpretation of data for complex ARGs structures such as arrangements of multiple copies of ARGs was possible (Li et al., 2018a, 2020), and it was recommended to perform multiple assemblies and single-molecule analysis to obtain reliable results. In another study, a novel KPC-2 variant, designated KPC-74, was found encoded in a plasmid in carbapenem-resistant *Klebsiella pneumoniae* (CRKP) by long-read sequencing, and conferred resistance to ceftazidime/avibactam and ertapenem, but is susceptible to imipenem and meropenem (Li C. et al.). In terms of AMR mechanism, bacterial DNA recombination system and tRNA upregulation under antibiotic stress rapidly provided antibiotic resistance at the early stage of bacterial growth (Fang et al.). Numerous structural variations were observed with nanopore sequencing after repairment of antibiotic-induced DNA breakage in the genome by the DNA recombination system.

ONT nanopore sequencing, QitanTech nanopore sequencing, and PacBio SMRT sequencing technologies are expanding the depth and width of AMR research. Although nanopore long-read length could reach up to 2Mbp with special genome extraction methods (Ammer-Herrmenau et al., 2021), N50 by traditional bacterial genome extraction methods is normally <50 kb (Li et al., 2018b; Ruh et al., 2021). As a result, the limitation of read length could also pose a challenge in deciphering the diversity and polymorphism of AMR genetic elements larger than 50 kb.

Although this Research Topic did not encompass all research articles on every aspect of the opportunities and challenges of long-read single-molecule sequencing technologies, the articles published here highlighted several important application directions in AMR research. Other fields such as the development of tools and analysis methods to decipher complex genomic structures of AMR bacteria, real-time genomic analysis, and tracking of AMR bacteria, and metagenomics of long-read sequencing data warrant more attention in the future. We thank all the authors and involved reviewers for their intelligence input, which shed light on the importance of incorporating long-read sequencing analysis into combating AMR bacteria comprehensively.

## Author Contributions

All authors listed have made a substantial, direct, and intellectual contribution to the work and approved it for publication.

## Funding

This work was supported by the Priority Academic Program Development of Jiangsu Higher Education Institutions (PAPD).

## Conflict of Interest

The authors declare that the research was conducted in the absence of any commercial or financial relationships that could be construed as a potential conflict of interest.

## Publisher's Note

All claims expressed in this article are solely those of the authors and do not necessarily represent those of their affiliated organizations, or those of the publisher, the editors and the reviewers. Any product that may be evaluated in this article, or claim that may be made by its manufacturer, is not guaranteed or endorsed by the publisher.
